# Toward earlier diagnosis and treatment of rare neurological disorders: the value of coordinated care and specialist centers

**DOI:** 10.3325/cmj.2019.60.156

**Published:** 2019-04

**Authors:** Paola Giunti, Stephen Morris, Maja Relja, Gregory Pastores, Vinciane Quoidbach

**Affiliations:** 1 University College London, London, UK; 2 Zagreb University School of Medicine, Zagreb, Croatia; 3 Mater Misericordiae University Hospital, Dublin, Ireland; 4 European Brain Council, Brussels, Belgium *vinc@braincouncil.eu*

Rare neurological diseases (RNDs) collectively exert a public health burden in terms of their manifestations severity and the total number of people afflicted across their lifespan. According to the European Reference Network on Rare Neurological Diseases, in Europe there are 500 000 people living with RNDs, while 60% of those affected are still undiagnosed due to significant phenotype and genotype heterogeneity in clinical presentation and disease course (1). Most rare disorders are of genetic origin. For many patients, considerable barriers exist in terms of access to appropriate care, delayed diagnosis, and treatment options. When patients are diagnosed, many are unable to access resources such as centers of expertise (or specialist centers), coordinated care, patient support systems, and effective treatment (2). Treatment of chronic RNDs has become increasingly multifaceted and comprises either disease-modifying drugs with different mechanisms of action, symptomatic therapies or other supportive therapies, or surgical procedures such as deep brain stimulation. Treatment must be highly customized to the needs of the individual.

A systematic review conducted in 2007 proposed the following working definition of care coordination: “the deliberate organisation of patient care activities between two or more participants (including the patient) involved in a patient's care, to facilitate the appropriate delivery of health care services.” This includes but is not limited to the use of specialist centers and specialist care coordinators (3). As recommended by the EU, each member state by the end of 2013 needs to develop coherent strategies for rare diseases to ensure that patients have access to high-quality care (4). Strategies such as National Rare Disease Plans include centers of expertise or specialist centers on rare diseases, integrated health care administrative databases, and national registries for rare diseases. While some countries coordinate their approach to rare disease management using comprehensive specialist centers, many countries do not, either because they have not yet adopted this approach or are employing different strategies (5). Multidisciplinary specialist centers directed to a particular RND may, in addition to the specialist neurologists and nurses, comprise geneticists, physiotherapists, occupational therapists, nutritionists, and neuropsychologists. These centers may even be cost-effective for the society by maintaining the patient’s ability to work and reducing the costs of home help and custodial care by keeping people with an RND independent or minimally so.

Motivated by addressing the existing gaps in care coordination, the European Brain Council, an organization promoting research on brain health and disorders in Europe, initiated in 2018 a two-year study on the value of early diagnosis and intervention, with an aim to assess the benefits of coordinated care and multidisciplinary care patterns on patient outcomes. The project, based on the conclusions of a previous EBC pan-European study (2017), The Value of Treatment for Brain Disorders – Policy White Paper (6), which called for a more seamless management of brain diseases, is now focusing on RNDs, aiming to include case studies on ataxia, dystonia, and phenylketonuria.

Ataxia and dystonia are rare movement disorders with broad heterogeneity, while phenylketonuria is an inherited metabolic disorder occurring due to deficiency of the enzyme phenylalanine hydroxylase. A lack of awareness and understanding of these RNDs among health care professionals makes their management challenging and highlights the importance of guidelines for diagnosis and treatment. For instance, although improving time to reach diagnosis and early intervention for ataxia and dystonia may be challenging, these improvements may ultimately reduce complications and disabilities. In the case of phenylketonuria, once a diagnosis is confirmed, usually through newborn screening programs, profound mental disability and severe neuro-psychiatric sequelae from the disease can be avoided by early intervention in the form of a “phenylalanine (Phe)-free” diet. However, this diet is highly restrictive, unpalatable, and can substantially affect patients’ and caregivers’ time and quality of life. As patients affected by phenylketonuria reach adolescence and adulthood, between 70 to 80% of them are not fully compliant with the prescribed diets (7). Overall, it is has not been evaluated so far whether better coordinated care for people with ataxia, dystonia, or phenylketonuria can be linked to measurable health gains, such as improved time to reach diagnosis, enhanced compliance with existing therapies, or better quality of life. Therefore, the aims of the ongoing study are to evaluate how the care for people with RNDs in Europe can be better coordinated and to assess the impacts of this on patient care and outcomes. The case studies analysis builds on complementary research exploring care pathways and economic evaluation being undertaken as part of the project.

To evaluate different models of coordinated care for RNDs, the study is developing a series of qualitative and quantitative benchmarks to identify treatment gaps and causal factors along the continuum of patient care in a pathway analysis. The study is also estimating the socioeconomic impact and health gains from specialist care centers for ongoing management of people with each disease. Data are taken from pre-existing surveys and diseases registries and previously published studies and are supplemented with new surveys to evaluate patient and family use and views of coordinated care. Coordinated care for RNDs is evaluated for each case study, involving research teams comprising clinical specialists, experts from the European Brain Council, and methodological expertise from academic partners including University College London, University of Zagreb School of Medicine, and University College Dublin. The research is undertaken in several European countries, depending on the availability of data, with an aim to capture variation in service provision. Evidence is assembled across the three case studies to produce policy recommendations.

The results of this research project are to be released by end 2020. We hope to analyze patient views and assess the cost-effectiveness of specialist centers for managing care of people with ataxia or dystonia in several European countries; and the cost-effectiveness of care coordinators for people with phenylketonuria to reduce drop-outs, encourage the compliance with the Phe-free diet, and improve overall health outcomes. Our aim is also to examine the role of national policy and programs, including National Rare Diseases Plans on the effective implementation of coordinated comprehensive services directed to ataxia, dystonia, and phenylketonuria.
